# Regional changes in brain apparent diffusion coefficient in fetuses with complex congenital heart disease and normal pregnancy assessed using diffusion-weighted imaging

**DOI:** 10.3389/fneur.2023.1136633

**Published:** 2023-05-30

**Authors:** Jia-Guang Song, Cong Sun, Mei Zhu, Jin-Xia Zhu, Nan Zhang, Guang-Bin Wang, Bin Zhao

**Affiliations:** ^1^Department of Ultrasound, Shandong Provincial Hospital, Shandong University, Jinan, Shandong, China; ^2^Department of Ultrasound, Shandong Provincial Hospital Affiliated to Shandong First Medical University, Jinan, Shandong, China; ^3^Department of Radiology, Beijing Hospital, National Center of Gerontology, Institute of Geriatric Medicine, Chinese Academy of Medical Sciences, Beijing, China; ^4^MR Collaboration, Healthcare Siemens Ltd., Beijing, China; ^5^Department of Radiology, Shandong Provincial Hospital Affiliated to Shandong First Medical University, Jinan, Shandong, China; ^6^Department of Radiology, Shandong Provincial Hospital, Shandong University, Jinan, Shandong, China

**Keywords:** brain, congenital heart disease, diffusion, fetus, regional changes

## Abstract

**Objectives:**

To explore changes in brain apparent diffusion coefficient (ADC) in normal fetuses and fetuses with complex congenital heart disease (CHD) during the second and early third trimesters.

**Methods:**

This single-center prospective study was conducted from May 2019 through October 2021. We measured and compared the mean ADC values between 23 fetuses with CHD and 27 gestational age (GA)-matched controls using covariance analyses. ADC density plots and histograms were used to compare brain characteristics. False-discovery rates (FDR, α = 0.05) correction was used for multiple testing.

**Results:**

The mean ADC in the frontal white matter, temporal white matter, parietal white matter, occipital white matter, cerebellar hemisphere, central area of the centrum semiovale, basal ganglia region, thalamus, and pons were not significantly different (all *p* > 0.05). Based on histogram analysis, there were no significant differences between the controls and fetuses with CHD after FDR correction. However, the ADC density plots showed significant heterogeneity between the controls and fetuses with CHD.

**Conclusion:**

The mean ADC values and ADC histogram analysis did not differ between the CHD and normal groups. The ADC density plots may provide supplementary information and improve the sensitivity for detecting early brain changes in fetuses with CHD.

## Introduction

Brain injury associated with complex congenital heart diseases (CHD) has been detected at an early stage before corrective surgery in neonates (1–4). Recent evidence suggests a high prevalence of structural brain anomalies (SBAs) in fetuses with different types of CHD (5). SBA rates were similar in the second and third trimesters of pregnancy (5). Dovjak et al. concluded that brainstem and cerebellar volumes on magnetic resonance imaging (MRI) are smaller in fetuses with CHD at 20–37 weeks of gestation (6). Some findings indicate that a small fetal brain volume may be a significant imaging biomarker of future neurodevelopmental risk in CHD (7).

Moreover, specific CHD lesions can cause abnormal cerebral blood flow (8–10) and chronic cerebral hypoxemia (11–14). Furthermore, they may influence neurogenesis and interneuron migration and cause delayed brain maturation and cortical development (15). However, the effects of volume changes, abnormal cerebral blood flow, and chronic cerebral hypoxemia on fetal brain diffusion are unclear during the second and early third trimesters.

Previous studies have shown that diffusion-weighted imaging (DWI) and apparent diffusion coefficient (ADC) images, based on microscopic water diffusion, can help quantify fetal brain maturation and reveal diffuse white matter abnormalities (16–18). For example, Miller et al. found abnormally high diffusion in the neonatal brain before corrective heart surgery (19). Other case reports have shown that ADC values in the periarterial white matter and thalamus were higher in fetuses with CHD during the late third trimester (20). However, the published studies only included a small sample size and the brain ADC changes in fetuses with CHD during the second and early third trimesters *in utero* remain unclear (20). In addition, no study has applied ADC density plots and ADC histograms to explore brain diffusion changes in fetuses with CHD. Therefore, exploration of brain diffusion changes during the early stages *in utero* is essential.

In this study, we explored brain ADC changes in normal fetuses and fetuses with complex CHD during the second and early third trimesters. We hypothesize that complex CHD affects fetal brain development at an early stage *in utero*, resulting in delayed brain maturation, structural abnormalities, and high ADCs compared to healthy gestational age (GA)-matched control fetuses.

## Materials and methods

### Participants

This prospective study was conducted from May 2019 through October 2021 in a single center. It was approved by our institutional review board and all participants provided written informed consent. We enrolled pregnant women with single fetuses showing complex CHD between 20 and 31 weeks of pregnancy and normal healthy fetuses between 20 and 40 weeks of pregnancy. All pregnant women underwent fetal brain DWI and no sedation or exogenous contrast agent was administered. The inclusion criteria for the normal group were routine pregnancy screening evaluations and no clinical or ultrasound evidence of other abnormalities. Our inclusion criterion for the CHD group was fetal echocardiogram confirmation of CHD according to established guidelines (21). The exclusion criteria for the normal and CHD groups were (a) maternal gestational diabetes mellitus, hypertensive disorder complicating pregnancy, (b) multiple pregnancies, (c) fetal chromosomal or genetic abnormalities diagnosed by amniocentesis, and (d) poor image quality due to fetal or maternal motion artifacts.

### MRI acquisition

All participants underwent prenatal brain MRI on a 3.0-T MR scanner (MAGNETOM Skyra, Siemens Healthcare, Erlangen, Germany) with an 18-channel body coil. DWI was performed using a single-shot echo-planar sequence and diffusion was measured in three orthogonal directions at two values of b (0 s/mm^2^ and 1,000 s/mm^2^). The parameters were as follows: repetition time (TR) = 4,900 ms; echo time (TE) = 87 ms; slice thickness = 4 mm without a slice gap; voxel size = 1.7×1.7×4.0mm ; and acquisition time = 2 min 12 s.

### Image analysis

**Figure 1:**
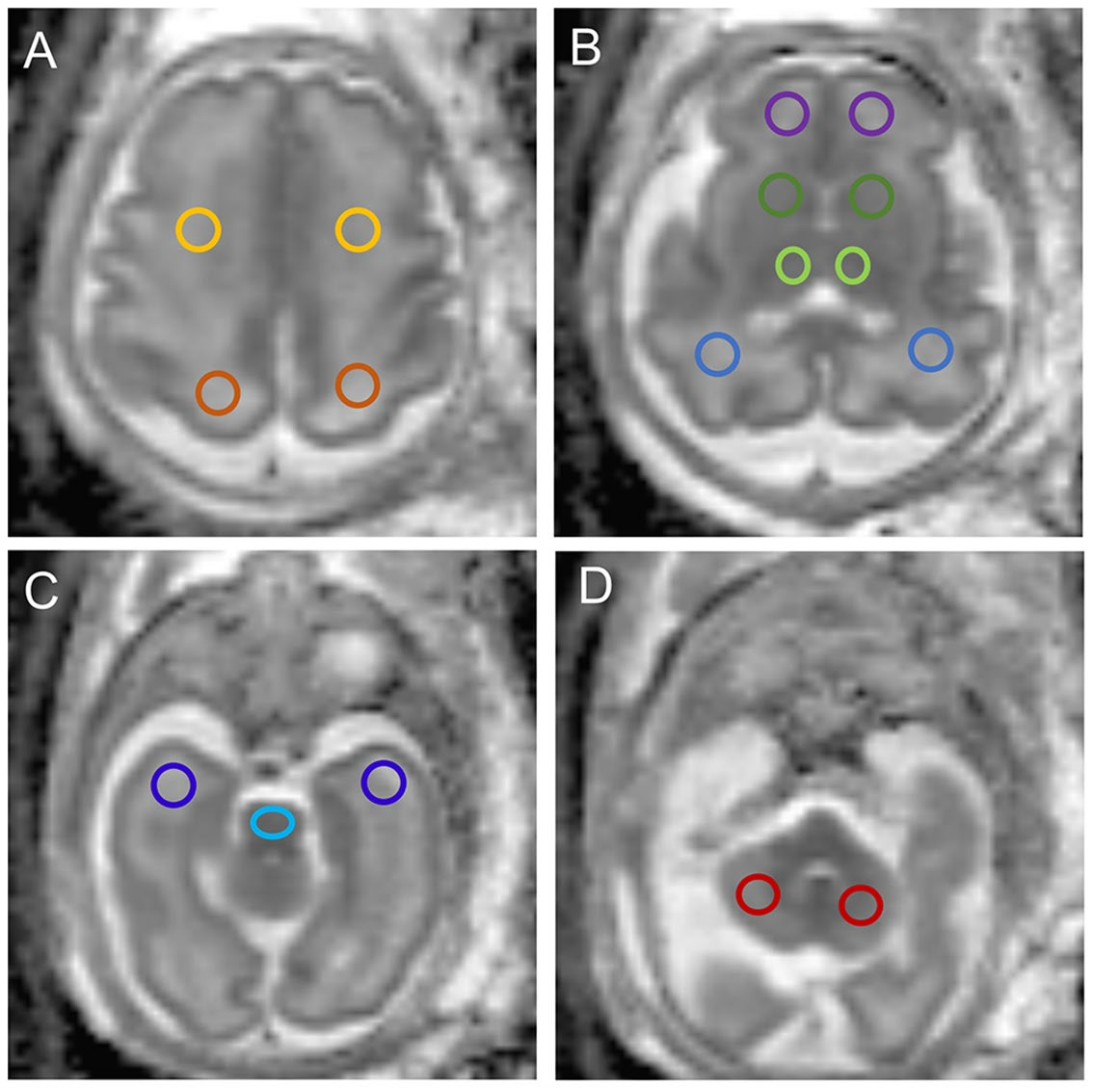
The ADC map of fetuses aged 30.71 weeks shows ROIs in the **(A)** centrum semiovale and parietal white matter (PWM); **(B)** frontal white matter (FWM), basal ganglia region (BGR), thalamus (TH), and occipital white matter (OWM); **(C)** temporal white matter (TWM) and pons; **(D)** cerebellar hemisphere.

Regions of interest (ROIs) were drawn on ADC maps in the frontal white matter (FWM), temporal white matter (TWM), parietal white matter (PWM), occipital white matter (OWM), cerebellar hemisphere (CH), central area of the centrum semiovale, basal ganglia region (BGR), thalamus (TH), and the pons (Figure 1). ROIs were placed by a pediatric neuroradiologist with 3 years of experience in fetal brain MR imaging. The neuroradiologist, who was blinded to group allocation, underwent a training session before placing the ROIs. The manually drawn ROIs varied in shape and size, depending on the specific brain region and fetal brain size. ADC values from both sides of the brain were averaged for each anatomic location.

### Histogram analysis

Single-slice ADC histograms were generated using Mazda software. Nine histogram parameters were obtained and statistically analyzed. The histogram shows the pixel frequency for each ADC value. The ADC map slice was chosen at this level and a single axial brain slice was selected because CHD can preferentially affect the basal ganglia and thalamus (20), as illustrated in Supplementary Figure S1A.

### Statistical analyses

Linear regression and polynomial quadratic nonlinear analyses were used to reveal the correlation between GA and ADCs in various brain regions. Density plots of ADCs in the nine ROIs for each GA were calculated using R version 4.0.2 (R Foundation, Vienna, Austria). These graphs represent the distribution of ADCs for each structure. With gestational age (GA) as a covariate, we conducted an analysis of covariance to compare ADC values in various brain regions between the CHD group and the normal control group. Histograms of ADCs in the whole brain for each GA were calculated. With gestational age (GA) as a covariate, we conducted an analysis of covariance to compare histogram parameters between the CHD group and GA-matched controls. False-discovery rates (FDR, α = 0.05) correction was used for multiple testing. Statistical analysis was performed using GraphPad Prism 9.0.0 (GraphPad Software, San Diego, CA, United States). Statistical significance was set at *p* < 0.05.

## Results

### Characteristics of the cohort

In this study, 23 pregnant women with a confirmed diagnosis of fetal complex CHD and 64 pregnant women with normal healthy fetuses were enrolled. In addition, we selected 27 GA-matched normal controls to compare ADCs with those obtained in CHD fetuses using covariance analysis. No significant difference was observed in GA between the CHD and the normal control groups (*p* = 0.38). The CHD structural lesions included complete transposition of great arteries (TGA), hypoplastic left heart syndrome (HLHS), coarctation of aorta (COA), tetralogy of Fallot (TOF), severe pulmonary stenosis or atresia (PS/PA), single ventricle (SV), and total anomalous pulmonary venous connection (TAPVC). The clinical characteristics of our cohort are shown in Table 1.

**Table 1:** Clinical characteristics in the CHD and GA-matcher groups.

	Control	CHD	*p* value
Maternal age(year)	29.78 ± 4.77	28.14 ± 3.43	0.18
GA (week)	26.81 ± 2.17	27.32 ± 1.87	0.38
Structural lesion			
TGA	NA	6 (26.1%)	
HLHS	NA	4 (17.4%)	
COA	NA	4 (17.4%)	
TOF	NA	5 (21.7%)	
PS/PA	NA	1 (4.3%)	
SV	NA	2 (8.6%)	
TAPVC	NA	1 (4.3%)	

NA indicates not applicable.

### Relationship between ADCs and GA in the normal group

**Figure 2:**
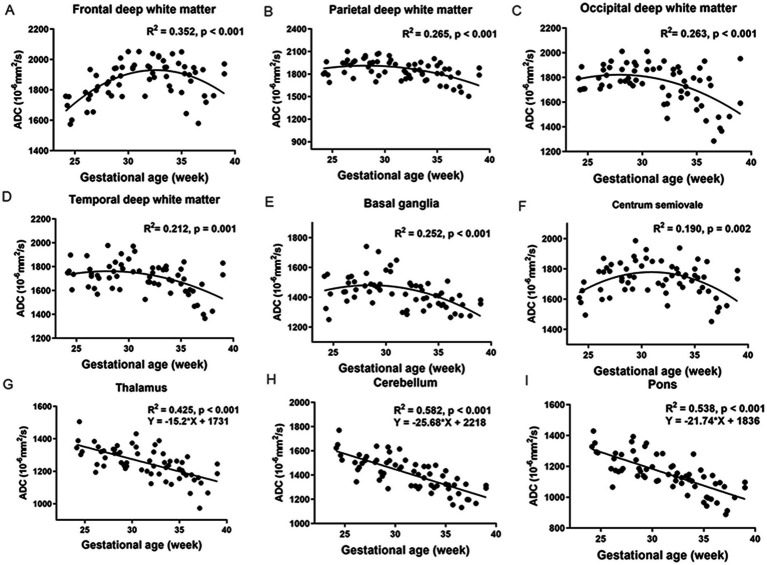
The relationship between ADCs and GA in the normal group (GA: 20–40 weeks) and their corresponding fitting curve. FWM **(A)**, PWM **(B)**, OWM **(C)**, TWM **(D)**, BGR **(E)**, Centrum semiovale **(F)**, TH **(G)**, CH **(H)**, Pons **(I)**.

Figure 2 shows the relationship between ADCs and GA in the normal group (GA: 20–40 weeks) and their corresponding fitting curves. A significant negative linear correlation was found in the CH (*R*^2^ = 0.582, *p* < 0.001), pons (*R*^2^ = 0.538, *p* < 0.001), and TH (*R*^2^ = 0.425, *p* < 0.001). A significant quadratic polynomial correlation was found in the FWM (*R*^2^ = 0.352, *p* < 0.001), PWM (*R*^2^ = 0.265, *p* < 0.001), OWM (*R*^2^ = 0.263, *p* < 0.001), TWM (*R*^2^ = 0.212, *p* = 0.001), BGR (*R*^2^ = 0.252, *p* < 0.001), and centrum semiovale (*R*^2^ = 0.190, *p* = 0.002).

**Figure 3:**
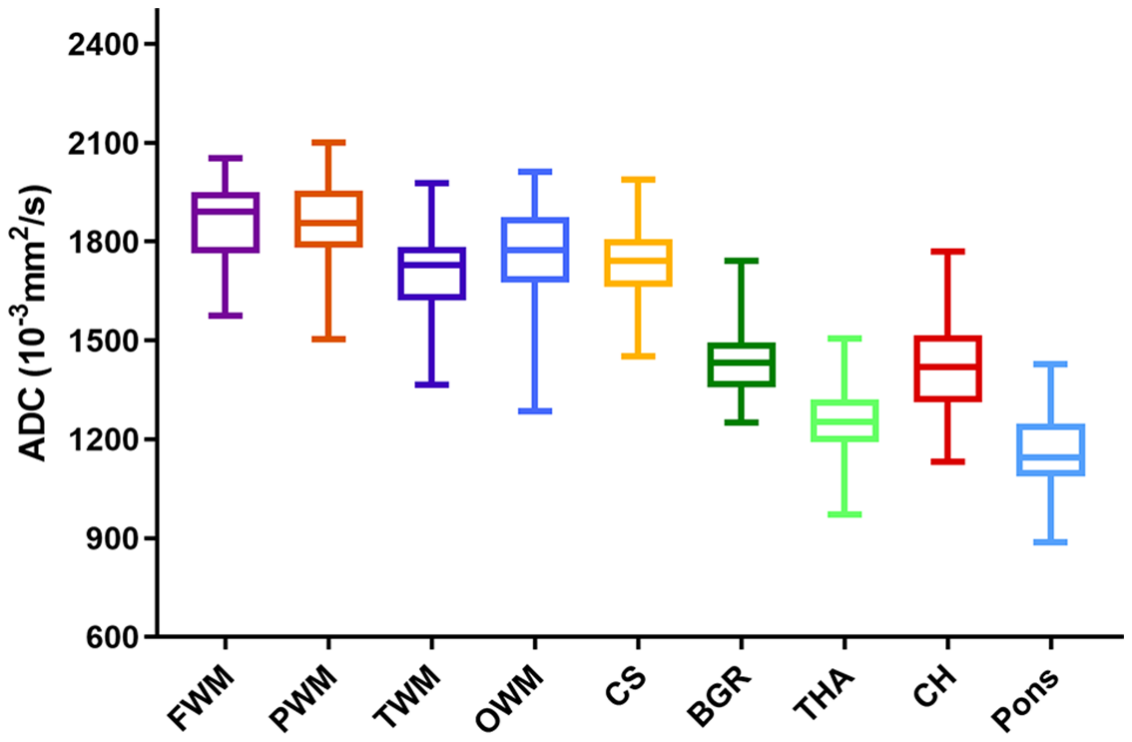
Regional brain ADCs in the normal group during the second and third trimesters.

The ADCs of each ROI showed significant heterogeneity and unique developmental trajectories, as depicted in Supplementary Figure S2. ADC density plots for GA 20–40 weeks in the nine representative ROIs in the normal group are summarized in Supplementary Figure S3. Figure 3 shows that the ADCs in the pons were the lowest, followed by TH and BGR, while those in PWM were the highest.

### Relationship between ADCs and GA in the CHD and GA-matched controls

**Figure 4:**
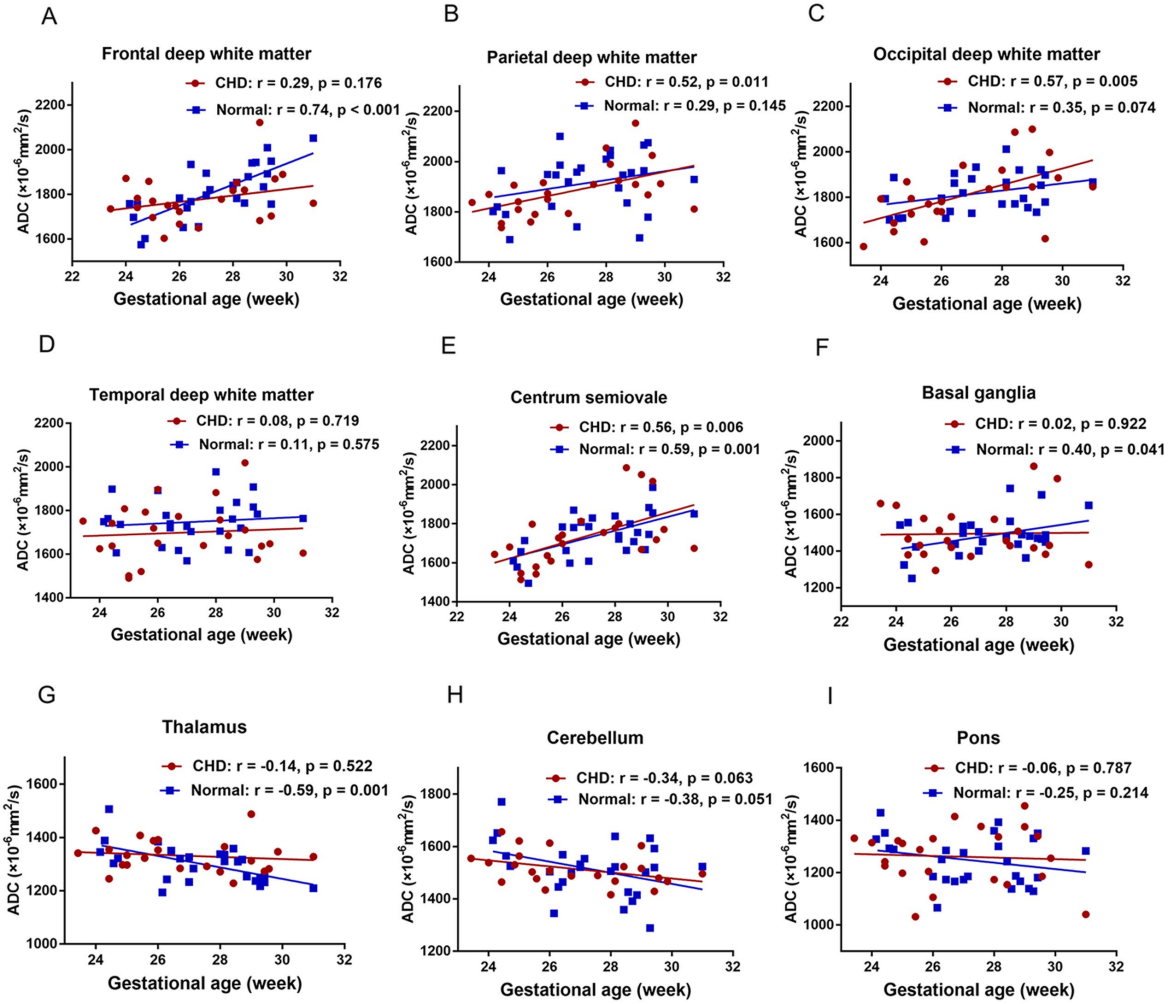
The relationship between ADCs and GA in the CHD and GA-matched control groups and their corresponding fitting curve. FWM **(A)**, PWM **(B)**, OWM **(C)**, TWM **(D)**, Centrum semiovale **(E)**, BGR **(F)**, TH **(G)**, CH **(H)**, Pons **(I)**.

Figure 4 shows the relationship between ADCs and GA in the CHD and GA-matched normal groups and their corresponding fitting curves. In the GA-matched normal group, the ADCs of the FWM, centrum semiovale, and BGR increased significantly across GA (*p* = 0.001, 0.001, and 0.041, respectively), and the ADCs of the TH decreased significantly across GA (*p* = 0.001). However, in the CHD group, the ADCs of the PWM, OWM, and centrum semiovale increased significantly across GA (*p* = 0.011, 0.005, and 0.006, respectively).

### Comparison of ADCs in various brain regions in fetuses with CHD and GA-matched controls

**Figure 5:**
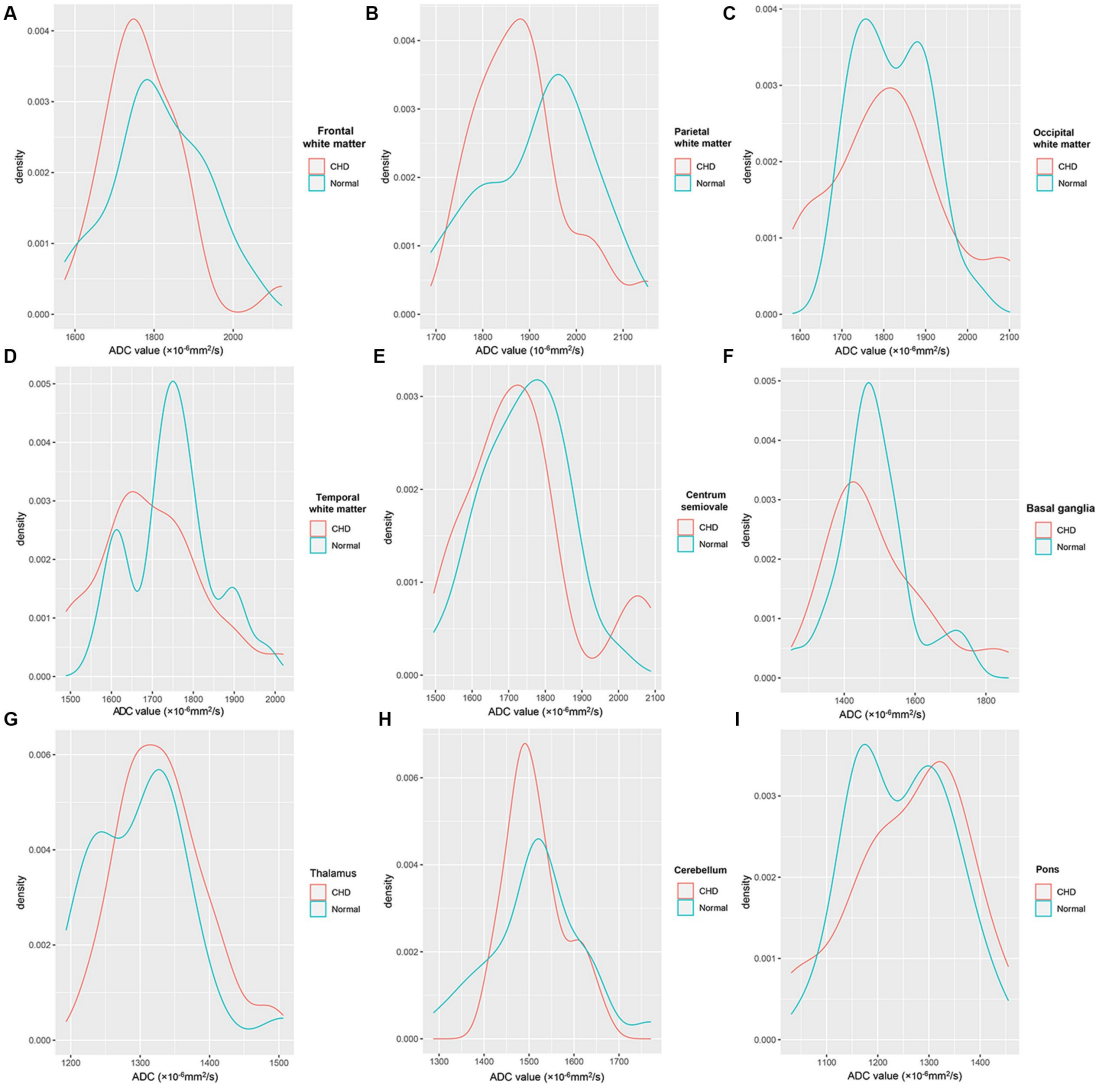
Density plots of the nine representative ROIs in the CHD and GA-matched control groups. FWM **(A)**, PWM **(B)**, OWM **(C)**, TWM **(D)**, Centrum semiovale **(E)**, BGR **(F)**, TH **(G)**, CH **(H)**, Pons **(I)**.

**Figure 6:**
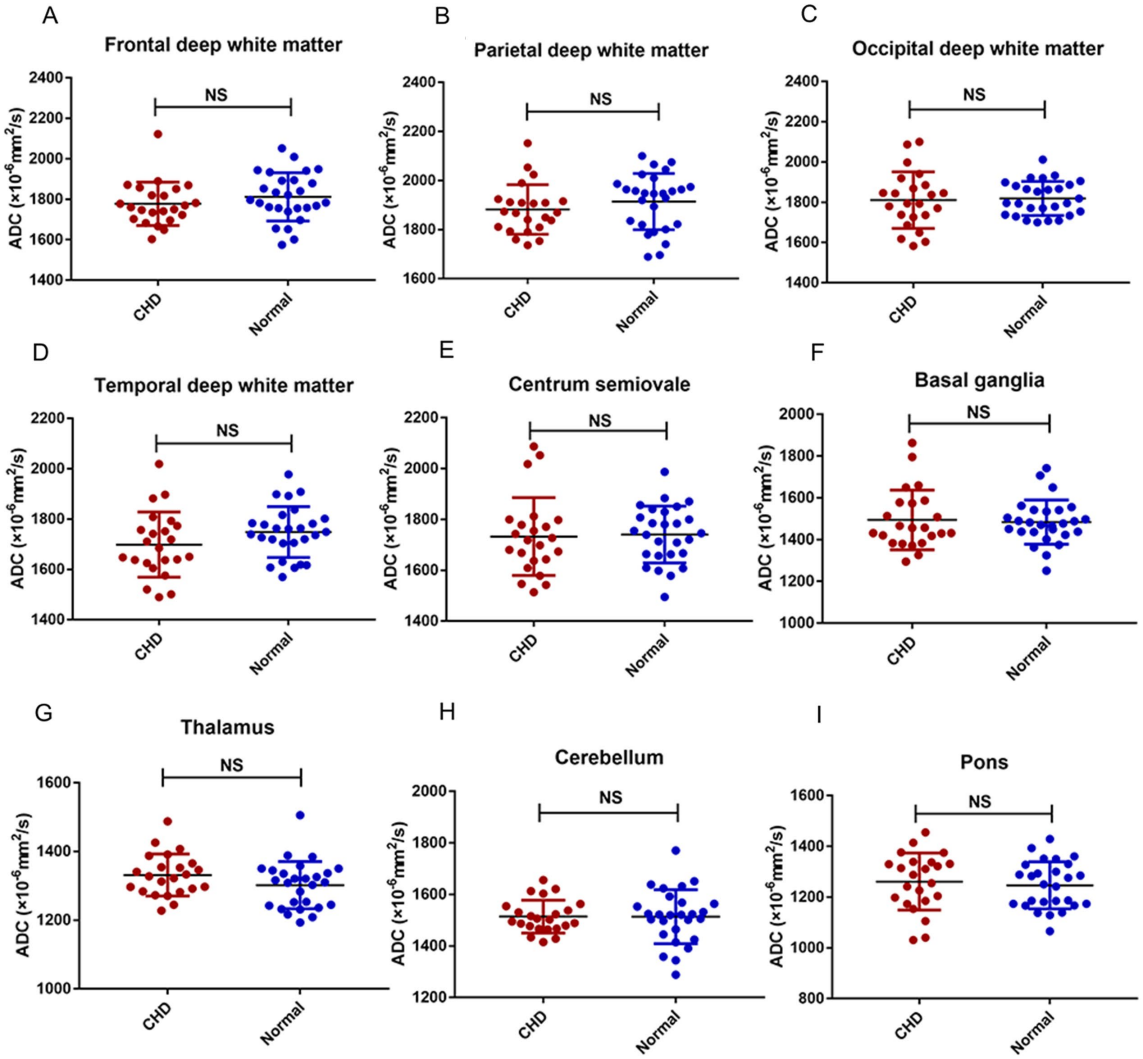
Comparison of ADCs in the CHD and normal GA-matched control groups. FWM **(A)**, PWM **(B)**, OWM **(C)**, TWM **(D)**, Centrum semiovale **(E)**, BGR **(F)**, TH **(G)**, CH **(H)**, Pons **(I)**.

Figure 5 shows the density plots of the nine representative ROIs in the GA-matched controls and CHD group, which also showed significant heterogeneity. Figure 6 shows a comparison of ADCs in the CHD group and GA-matched controls. ADCs were not significantly different between the FWM, TWM, PWM, OWM, CH, centrum semiovale, BGR, TH, and pons (*p* > 0.05).

### ADC histogram analysis in the CHD group and GA-matched controls

Table 2 shows a comparison of all histogram features between the two groups. Among the characteristics of the nine parameters extracted from the histogram, the difference in the 10th percentiles between the two groups was significant (*p* = 0.046). However, it showed no statistical significance after FDR correction. In addition, the mean, skewness, kurtosis, variance, minimum and maximum values, and 50th, 90th, and 99th percentiles showed no differences (all *p* > 0.05). Representative cases of histogram features are shown in Supplementary Figures S1B,C.

**Table 2:** Comparison of all histogram features between the two groups.

	Control	CHD	*p* value
Mean (10^−6^ mm^2^/s)	1604.77 ± 57.22	1618.4 ± 138.32	0.102
Skewness	1.0688 ± 0.3280	0.8503 ± 0.4432	0.091
Kurtosis	2.2688 ± 1.0661	1.6106 ± 0.9521	0.064
pera1 (10^−6^ mm^2^/s)	1144.22 ± 76.97	1144.50 ± 146.56	0.065
pera10 (10^−6^ mm^2^/s)	1313.52 ± 64.04	1330.10 ± 134.05	0.046
Pera50 (10^−6^ mm^2^/s)	1568.87 ± 58.25	1588.50 ± 131.42	0.057
Pera90 (10^−6^ mm^2^/s)	1922.13 ± 84.44	1932.10 ± 184.19	0.510
Pera99 (10^−6^ mm^2^/s)	2485.91 ± 196.77	2424.95 ± 340.60	0.486
Variance	71043.48 ± 21746.55	70410.75 ± 34286.30	0.177
Min	987.04 ± 113.56	897.90 ± 167.77	0.176
Max	2800.30 ± 253.22	2704.40 ± 305.40	0.405
SD	261.60 ± 41.87	256.05 ± 60.99	0.328

## Discussion

Our results show that ADCs of healthy fetuses acquired with a 3.0-T scanner align with those reported in previous studies (22–25). In addition, this *in vivo* study suggests that the mean ADCs in the CHD group did not differ significantly from those in the GA-matched normal fetuses during the second and early third trimesters. In addition, we applied ADC histogram analysis for the first time in the brains of fetuses with CHD at GA 20–30 weeks.

Our results demonstrate that regional brain ADC measurements using 3.0-T scanners are feasible. With the widespread application of 3.0-T MRI in the fetal brain, establishing normal ADC references to evaluate normal and abnormal brain development quantitatively has gained importance. In our study, the ADCs differed among various brain regions, consistent with previously reported values for 1.5-T scanners (22, 23, 26). Regional ADC differences can reflect cellularity density, neuronal maturation, and myelination during gestation in brain development. For example, in most deep white matter areas, the primary increase before the 30^th^ gestational week could be due to the cellular structure and intermediate zone containing migrating cells (27). The subsequent decline results from the disappearance of the intermediate zone, a decrease in water content, and maturation.

In contrast, the pons showed the lowest ADC, followed by TH, BGR, and CH. This may be because these regions are composed of more densely packed cells than non-myelinated white matter, contain larger interstitial water, and undergo much earlier maturation (26). In addition, the process of maturation and myelination of deep brain white matter in a normal fetal brain is from the inferior to the superior and from the posterior to the anterior (28, 29).

In contrast to earlier findings (20, 30), no difference was detected in the mean ADCs of the FWM, TWM, PWM, OWM, CH, centrum semiovale, BGR, TH, and pons between the CHD and GA-matched controls. Although this finding is inconsistent with our hypothesis, it can be explained in several ways. First, the fetuses with CHD that we included were between the second and early third trimesters; this early period may include fetal brain sparing and autoregulation (10). Second, the CHD types we included were heterogeneous, and different types of CHDs may have varied effects on brain maturation. Third, ROI placements are usually over a small brain area, which may introduce a subjective component to image measurements and analysis as well as some bias.

We utilized ADC histograms to compare brain characteristics between fetuses with CHD and normal fetuses. After applying the FDR correction, the ADC histogram analysis did not reveal any information in early-stage fetuses with CHD. In contrast to histograms, density plots offer a smoother representation and exhibit less sensitivity to bin size and quantity. When outliers exist, their proportion in the sample can be easily visualized through the corresponding area under the curve in the density plot. This feature is advantageous when addressing bimodal or multimodal distributions, which may indicate a heterogeneous sample (31). ADC density plots primarily focus on data distribution, making them less prone to SNR and other scanning-related issues. When we introduced density maps to compare brain characteristics between fetuses with CHD and normal fetuses, we observed significant heterogeneity between the CHD and normal control groups. These results suggest that ADC density plots can provide more comprehensive differentiation information, enhancing the sensitivity in detecting early fetal changes in fetuses with CHD.

This study has some limitations. First, our research was a single-center study and the sample size of the CHD group was relatively small. Second, we did not classify complex CHD into subgroups because of the small number of cases and could not obtain a specific conclusion in each category. Third, in this study, we only aimed to explore the brain diffusion changes in fetuses with CHD at 20–31 weeks of gestation. Lastly, we did not include fetal sex as a covariate in our analysis, even though no current literature suggests that fetal sex has an impact on intracranial ADC values. In the future, we will include more late-pregnancy fetuses, neonates, and infants to explore brain development in a longitudinal study.

## Conclusion

We used DWI to quantify the ADCs in fetuses across GA during the second and third trimesters. The mean ADCs and histogram analysis in the complex CHD group were not significantly different from those in GA-matched normal fetuses during the second and early third trimesters. However, the ADC density plots showed significant heterogeneity between controls and fetuses with CHD, and thus, may provide supplementary information and improve the sensitivity for detecting early brain changes in fetuses with CHD.

## Data availability statement

The original contributions presented in the study are included in the article/Supplementary material, further inquiries can be directed to the corresponding author.

## Ethics statement

The studies involving human participants were reviewed and approved by Ethics Committee of Shandong Provincial Hospital. The patients/participants provided their written informed consent to participate in this study.

## Author contributions

J-GS, CS, G-BW, and BZ conceived the idea and conceptualised the study. J-GS, CS, MZ, J-XZ, and NZ collected the data. J-GS, CS, J-XZ, MZ, and NZ analysed the data. J-GS and CS drafted the manuscript. G-BW and BZ reviewed the manuscript. All authors contributed to the article and approved the submitted version.

## Funding

This study was supported by the key R&D Program of Shandong Province (2022CXGC010504) and (2018GSF118112).

## Conflict of interest

J-XZ was employed by MR Collaboration, Healthcare Siemens Ltd.

The remaining authors declare that the research was conducted in the absence of any commercial or financial relationships that could be construed as a potential conflict of interest.

## Publisher’s note

All claims expressed in this article are solely those of the authors and do not necessarily represent those of their affiliated organizations, or those of the publisher, the editors and the reviewers. Any product that may be evaluated in this article, or claim that may be made by its manufacturer, is not guaranteed or endorsed by the publisher.

## Supplementary material

SUPPLEMENTARY FIGURE S1 Representative cases of histogram features. **(A)** The ROI of the histogram; **(B)** A case of CHD at the gestaional age of 25w + 4; **(C)** A case of normal fetus at the gestaional age of 25w + 2.

SUPPLEMENTARY FIGURE S2 Density plots of the nine representative ROIs of ADCs across gestational ages (20-40 weeks). ADC = apparent diffusion coefficient.

SUPPLEMENTARY FIGURE S3 Density plots of ADCs at different gestational ages in the normal group’s nine representative ROIs. **(A)** FWM, **(B)** PWM, **(C)** OWM,**(D)** TWM, **(E)** Centrum semiovale, **(F)** BGR,**(G)** TH,**(H)** CH, **(I)** Pons.
