# Supplementary figures and images for: Regional changes in brain apparent diffusion coefficient in fetuses with complex congenital heart disease and normal pregnancy assessed using diffusion-weighted imaging
Source: Front Neurol. 2023 May 30;14:1136633. doi: 10.3389/fneur.2023.1136633 (PMC10283352; doi:10.3389/fneur.2023.1136633)

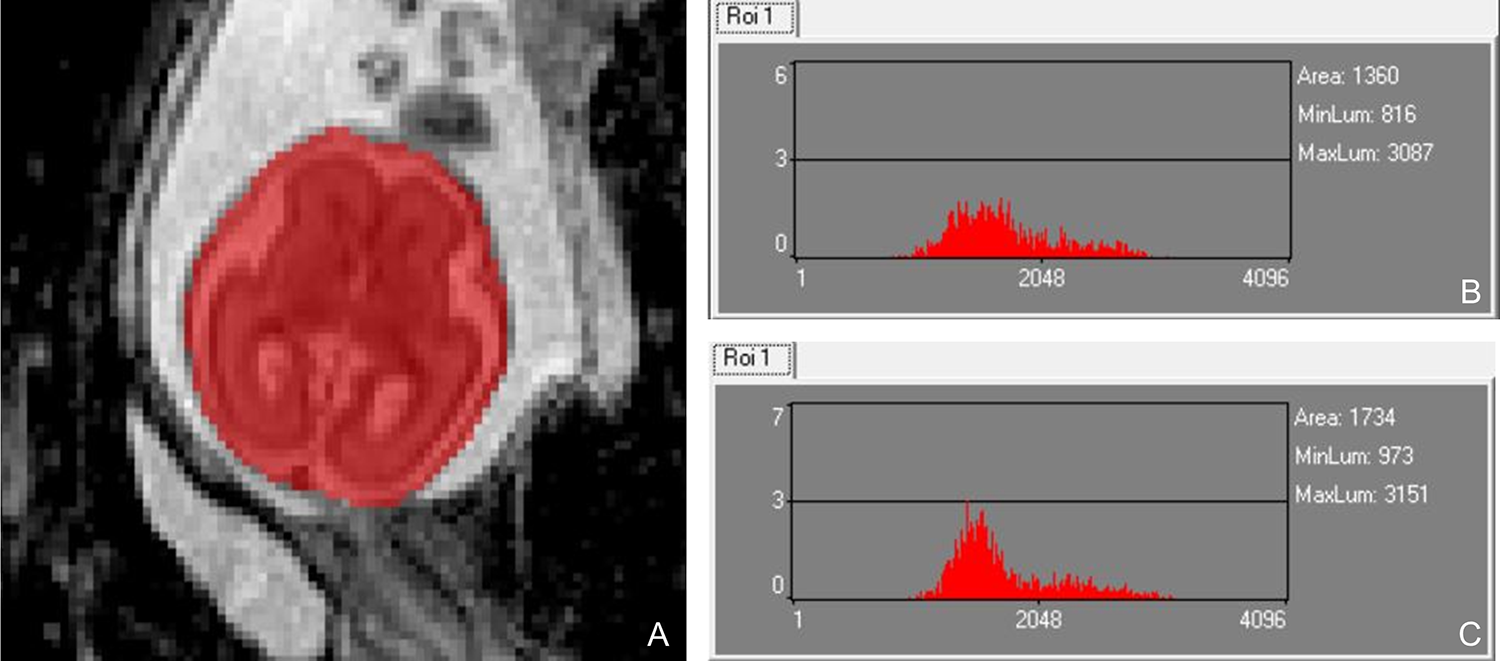

Supplement: SUPPLEMENTARY FIGURE S1 — Representative cases of histogram features. (A) The ROI of the histogram; (B) A case of CHD at the gestaional age of 25w + 4; (C) A case of normal fetus at the gestaional age of 25w + 2. [file Image_1.TIF]

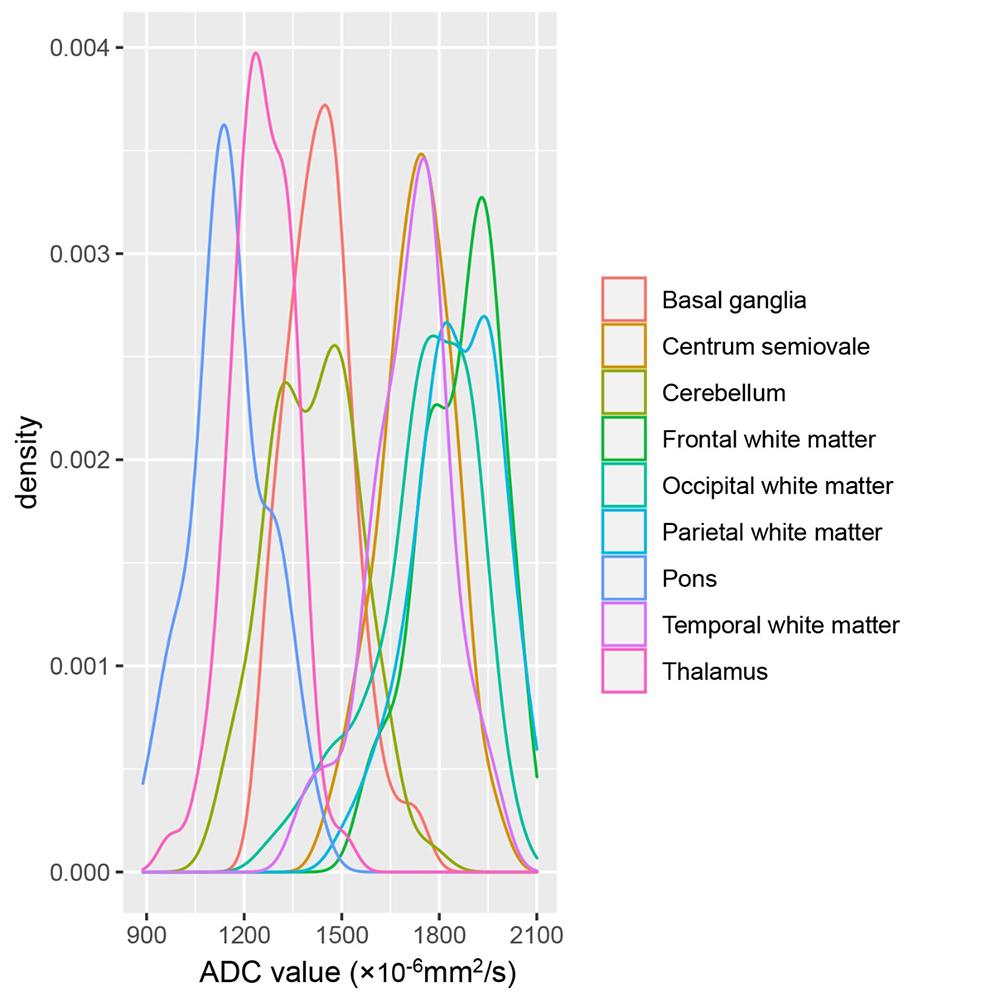

Supplement: SUPPLEMENTARY FIGURE S2 — Density plots of the nine representative ROIs of ADCs across gestational ages (20-40 weeks). ADC = apparent diffusion coefficient. [file Image_2.TIF]

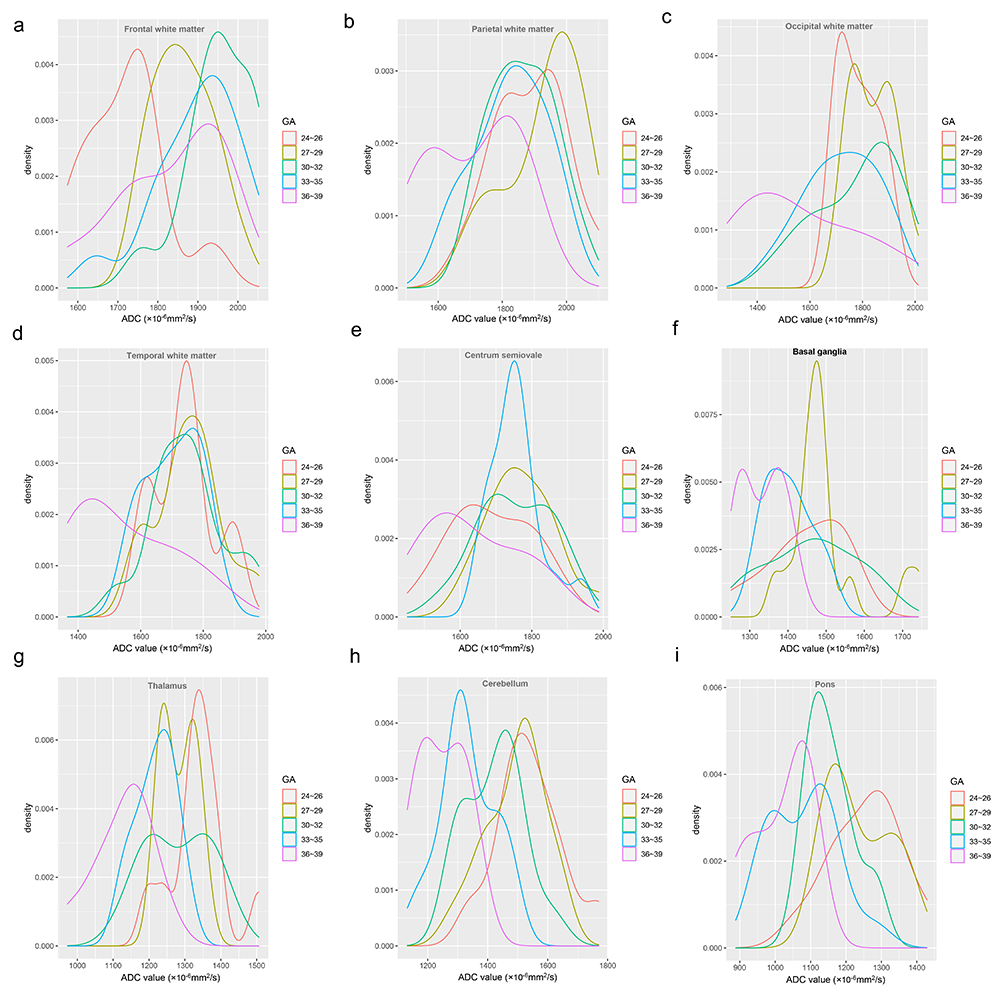

Supplement: SUPPLEMENTARY FIGURE S3 — Density plots of ADCs at different gestational ages in the normal group’s nine representative ROIs. (A) FWM, (B) PWM, (C) OWM,(D) TWM, (E) Centrum semiovale, (F) BGR,(G) TH,(H) CH, (I) Pons. [file Image_3.TIF]
